# Intercalation of Li^**+**^ vs Na^**+**^ Ions in AlOOH Thin Films: Toward Low-Cost Solid-State Electrolytes

**DOI:** 10.1021/acsomega.5c04820

**Published:** 2025-07-21

**Authors:** Martín A. Ruiz-Molina, Leunam Fernandez-Izquierdo, Rubén O. Grijalva-Saavedra, Manuel Quevedo-López, Mérida Sotelo-Lerma

**Affiliations:** † Departamento de Investigación en Polímeros y Materiales, Universidad de Sonora, Blvd. Luis Encinas y Rosales S/N, Hermosillo, Sonora 83000, Mexico; ‡ Materials Science and Engineering Department, University of Texas at Dallas, 2601 North Floyd Road, RL 10, Richardson, Texas 75080, United States

## Abstract

This study explores the influence of ionic incorporation via immersion in LiCl and NaCl on the structural, morphological, and electrochemical properties of AlOOH thin films synthesized by chemical bath deposition. X-ray diffraction reveals that LiCl treatment induces the formation of lithium carbonate and aluminum hydroxide phases, a transformation absent in NaCl-treated films, which retain the pristine AlOOH structure. Scanning electron microscopy shows pronounced nanoflake growth in LiCl-treated films, while NaCl exposure yields negligible morphological changes. X-ray photoelectron spectroscopy confirms the formation of surface carbonate species in LiCl-treated samples, suggesting enhanced CO_2_ adsorption and conversion facilitated by Li^+^. Electrical characterization via current–voltage measurements indicates increased conductivity in LiCl-treated films due to Li^+^ mobility, contrasting with the resistive nature of NaCl-treated counterparts. Electrochemical impedance spectroscopy further supports these findings, revealing ionic diffusion behavior in LiCl-treated samples and limited ionic transport in the NaCl-treated films. These results highlight the unique role of Li^+^ in the modification of AlOOH thin films, yielding a room temperature ionic conductivity of 0.77 × 10^–4^ S cm^–1^, underscoring their potential for applications in ionic sensing, electrochemical storage, and gas capture technologies.

## Graphical abstract

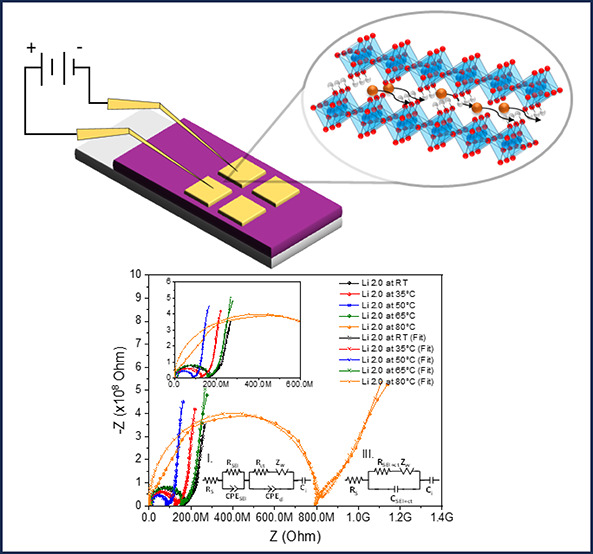

## 1 Introduction

The development of materials for solid-state battery applications has become a priority in the fields of materials science and energy engineering. Conventional batteries, such as lithium-ion batteries with liquid electrolyte, present multiple challenges that limit their safety and efficiency, including the use of flammable materials, the risk of explosion due to overheating, and the formation of metallic dendrites that can cause internal short circuits. These problems have prompted the search for safer, longer-lasting, and more energy-dense technologies, among which solid-state batteries represent one of the most promising alternatives.^1–3^

One of the keys to the development of solid-state batteries lies in the implementation of stable solid electrolytes with high ionic conductivity, a wide electrochemical stability range, and good compatibility with the electrodes. Unlike liquid electrolytes, solid electrolytes can offer greater thermal and mechanical safety, reducing the risk of leakage or ignition. In addition, their design allows for the development of more compact devices, such as microbatteries for portable biomedical applications or integrated into flexible electronic systems.^4–8^

The materials that have been explored as solid ceramic electrolytes are conductive lithium oxides, such as Li_3.3_La_0.56_TiO_3_, LiTi­(PO_4_)_3_, LiPON, Y_2_O_3_–ZrO_2_, Al_2_O_3_, γ-LiAlO_2_, β-Al_2_O_3_, and LiOH/Al­(OH)_3_,^9–12^ and phosphate-based composite materials or perovskites. These can be obtained by different methods, such as solid-state synthesis, sol–gel, and deposition techniques, such as sputtering or pulsed laser deposition (PLD), which allow one to obtain thin films with adjustable properties according to the desired application.

In this context, the material AlOOH (boehmite), doped with cations such as Li^+^ or Na^+^, has emerged as an interesting candidate for study as a thin-film electrolyte. Its lamellar structure and the possibility of modifying its surface by chemical treatments make it a potentially useful system for applications in microbatteries, where materials with good thermal stability, controlled dielectric properties, and pathways for ionic migration at the nanoscale are required.

This study aims to address the development of the synthesis of the material AlOOH (boehmite) by chemical solution methods, with the objective of incorporating monovalent cations such as Li^+^ or Na^+^ within its structure. The research focuses on obtaining homogeneous thin films with morphological and structural properties suitable for their potential application as a solid electrolyte in solid-state microbatteries. The incorporation of these cations could enhance the ionic conduction properties of the material, as well as influence its thermal and chemical stability—key factors for its electrochemical performance. In addition, the aim is to implement a low-cost synthesis route that minimizes energy consumption and the generation of hazardous waste, in line with the principles of green chemistry. This approach not only allows the development of functional materials with technological impact but also contributes to establishing sustainable and scalable processes for applications in next-generation energy devices.

## 2 Experimental Section

For this project, two approaches were contemplated: (i) *in situ* synthesis, one step using chemical bath solution (CBD), and (ii) two-step synthesis, first the synthesis of the AlOOH film by CBD and then dip coating Li^+^ and Na^+^ salts added in solution.

### 2.1 Reagents and Materials

Aluminum sulfate 0.5 M (FAGA LAB) as the source of the metal ion Al^3+^, triethanolamine (TEA) 50% (CTR SCIENTIFIC) as the metal ion complexing agent, ammonium chloride and ammonium hydroxide to form the buffer pH 10 (99.9% purity, Fermont, and 29.0% purity as NH_3_, Fermont, respectively) as the pH regulator of the reaction, and lithium chloride and sodium chloride 1.0 M (both with 99.9% purity, Fermont) as sources of cations. The total volume of the reaction mixture was 100 mL for both methods. The substrates used were glass (PEARL MICROSCOPE SLIDES 25.4 mm × 76.2 mm × 1.0 mm), which were cleaned with Alconox detergent and distilled water and stored in a sample box until ready for use.

### 2.2 Chemical Bath Deposition Method

The synthesis was carried out in a LAUDA thermo bath at a temperature of 70 °C with deposition times of 0.5, 1.0, and 2.0 h. In a beaker, first 5 mL of buffer pH 10 and 10 mL of triethanolamine were added at the same time, stirring lightly by hand, and then the aluminum sulfate at 0.5 M aluminum sulfate diluted 3 mL in 20 mL of distilled water was added in the same way and shaken lightly for 10 s until a homogeneous dissolution occurs. Finally, 1 mL of the lithium chloride or sodium chloride solution was added, and distilled water was added until 100 mL is completed.

After the time of deposition, the substrates are removed from the beaker. They are then washed with a direct jet of distilled water to remove the supernatant and terminate the film’s growth, sliding smoothly into cotton previously humidified with distilled water. They are left to dry at room temperature.

### 2.3 Dip Coating Method

First, the synthesis of the AlOOH material was performed, where 3 mL of aluminum sulfate, 5 mL of buffer pH 10, and 10 mL of TEA were used, following the previous steps of Section 2.2, leaving a deposition time of 120 min. After the elapsed time, the reaction substrates were removed and rinsed with distilled water and wet absorbent cotton, as in the previous section.

A 5 mL portion of 1 M lithium chloride or sodium chloride was used for the next step. The thermos bath was used at a temperature of 70 °C, leaving immersion times of 0.5, 1.0, and 2.0 h. After the elapsed time, the substrates were removed, rinsed with distilled water and wet cotton, and left to dry at room temperature.

### 2.4 Conductivity Measurement Geometry in Thin Films

The configuration used was as follows: the films were deposited on glass substrates using the methods described above. The gold contacts were deposited using an e-beam evaporator with a thickness of 200 nm. The contact pattern consisted of 240 × 240 μm squares, 310 μm apart.

The determination of the ionic conductivity in in-plane configuration for the materials studied was carried out from the approach of eq 1,^13,14^ which served as a theoretical basis for the treatment of the experimental data obtained.

1 $$\sigma_{\mathrm{in}\;\mathrm{plane}}=\frac{L}{Rx\left(lxd\right)}$$

where σ_in plane_: ionic conductivity in-plane, S cm^–1^; *L*: contact separation, cm; *R*: charge transfer resistance, Ω; *l*: electrode length, cm; *d*: sample thickness, cm.

### 2.5 Characterization Techniques

Grazing incidence X-ray diffraction (GIXRD) measurements were performed using Rigaku SmartLab X-ray diffractometer equipment, employing a Cu Kα source (λ = 1.54 Å) at 0.2 degrees. Fourier transform infrared spectroscopy (FTIR) model PerkinElmer FT-IR/FIR Spectrometer Frontier MIR zone. X-ray emitted photoelectron spectroscopy (XPS) analysis employing PERKIN ELMER PHI 5600 ESCA System equipment with a monochromatic Al Kα 1486.7 eV source used the main C peak as a binding energy reference of 284.8 eV. Surface and cross-sectional micrographs were performed by a ZEISS Supra 40 scanning electron microscope (SEM). The gold tips of the Cascade Microtech four-probe station and the CH Instruments Electrochemical Workstation CHI760E were used for IV curves and electrochemical impedance spectroscopy characterization.

## 3 Results and Discussions

Figure 1a shows the crystal structure of the reference material. The XRD characterization at a grazing angle showed that the film, as deposited to obtain aluminum hydroxide, resulted in a phase called boehmite (γ-AlOOH) which is obtained taking into account the deposition time, pH reaction solution, and the slow growth control of the film.^15–17^ The important signals to identify this phase are found at 38.45, 45.65, 48.69, and 49.32° 2θ with planes of (031), (131), (051), and (200), corresponding to the crystallographic chart PDF 01-083-2384. Although the strongest reflection of γ-AlOOH at ∼14.5° 2θ (020) is typically the most intense, it is not observed in the sample due to both the attenuation caused by the glass substrate in the 10–20° 2θ range^18,19^ and the use of a grazing incidence angle (ω = 0.2°), which suppresses low-angle reflections. Phase identification was confirmed using higher-angle peaks (e.g., (031), (131), (051), and (200)), consistent with PDF 01-083-2384.

**Figure 1:**
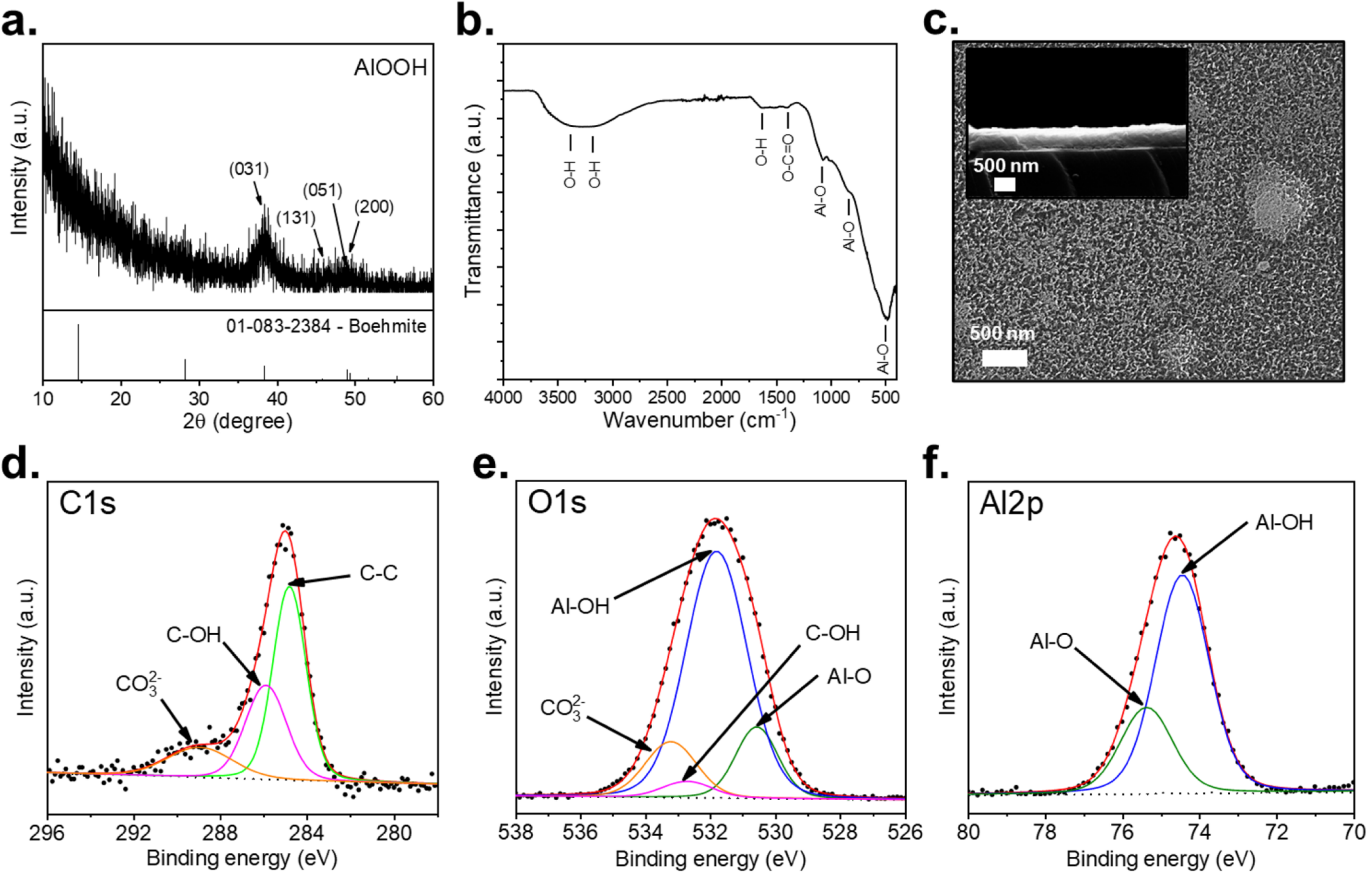
(a) XRD pattern of the chemical bath-deposited film; (b) FTIR spectra of the chemical bath-deposited film; (c) SEM micrograph in the top view of the deposited film and cross-section as an inset; and (d–f) XPS spectra of the C 1s, O 1s, and Al 2p orbitals of the AlOOH deposited film.

Analysis by FTIR spectroscopy, shown in Figure 1b, reveals the characteristic spectrum of the AlOOH film. Two prominent vibrational modes are identified around 3395 and 3181 cm^–1^,^20,21^ which are attributed to vibrations of the O–H bonds from hydroxyl groups of AlOOH and water. Additionally, bands are observed at 1632 cm^–1^,^22^ associated with the presence of water molecules adsorbed on the film surface, corresponding to vibrations of the H–O–H bonds. The band recorded around 1398 cm^–1^ is related to vibrations of the carbonate group (CO_3_^2–^).^23^ Finally, the signals at 1081, 839, and 501 cm^–1^ are assigned to vibrations characteristic of tetrahedral and octahedral structures.^22,24–26^ Using SEM in Figure 1c, the surface morphology of the AlOOH film was obtained, which illustrates porosity thanks to the interconnection of nanowafers^27,28^ and clusters coming from the reaction growth mechanism. In addition, the SEM cross-section shown in the inset helped to determine the thickness of the material, which is 585 nm. On the other hand, the XPS spectra in Figure 1d–f reveal the composition of the AlOOH film with the C 1s, O 1s, and Al 2p orbitals. Using the C 1s orbital at the beginning, it is shown as surface carbon, which is not part of the sample but is adsorbed by the environment. On the other hand, the contributions of Al–OH and Al–O are evident in the O 1s orbital, about 531.80 eV and about 530.56 eV, respectively, and in the Al 2p orbital, about 74.43 eV and about 75.35 eV, respectively.^29–31^

Although detailed elemental profiling across the film thickness was not performed, the uniform morphology observed in the SEM cross-sections, along with the consistent ionic conductivity and increasing XPS signals with immersion time, supports the interpretation of homogeneous cation incorporation throughout the AlOOH films, as will be discussed in the following sections.

### 3.1 XRD Analysis

The results obtained using the dip coating methodology showed new peaks along the different immersion times, which are represented by the main peak of the crystallographic chart mp-1236333 around 11.61° 2θ, followed by the peak around 35.7° 2θ with the planes (002) and (310). On the other hand, the signals found in Figure 2a for the Li 1.0 h and Li 2.0 h diffractograms, around 11.61, 18.86, and 23.33° 2θ with planes in (002), (101), and (004), are due to the compound [LiAl_2_(OH)_6_]_2_CO_3_ presented by Ashto,^9^ which was obtained by solution reaction.

**Figure 2:**
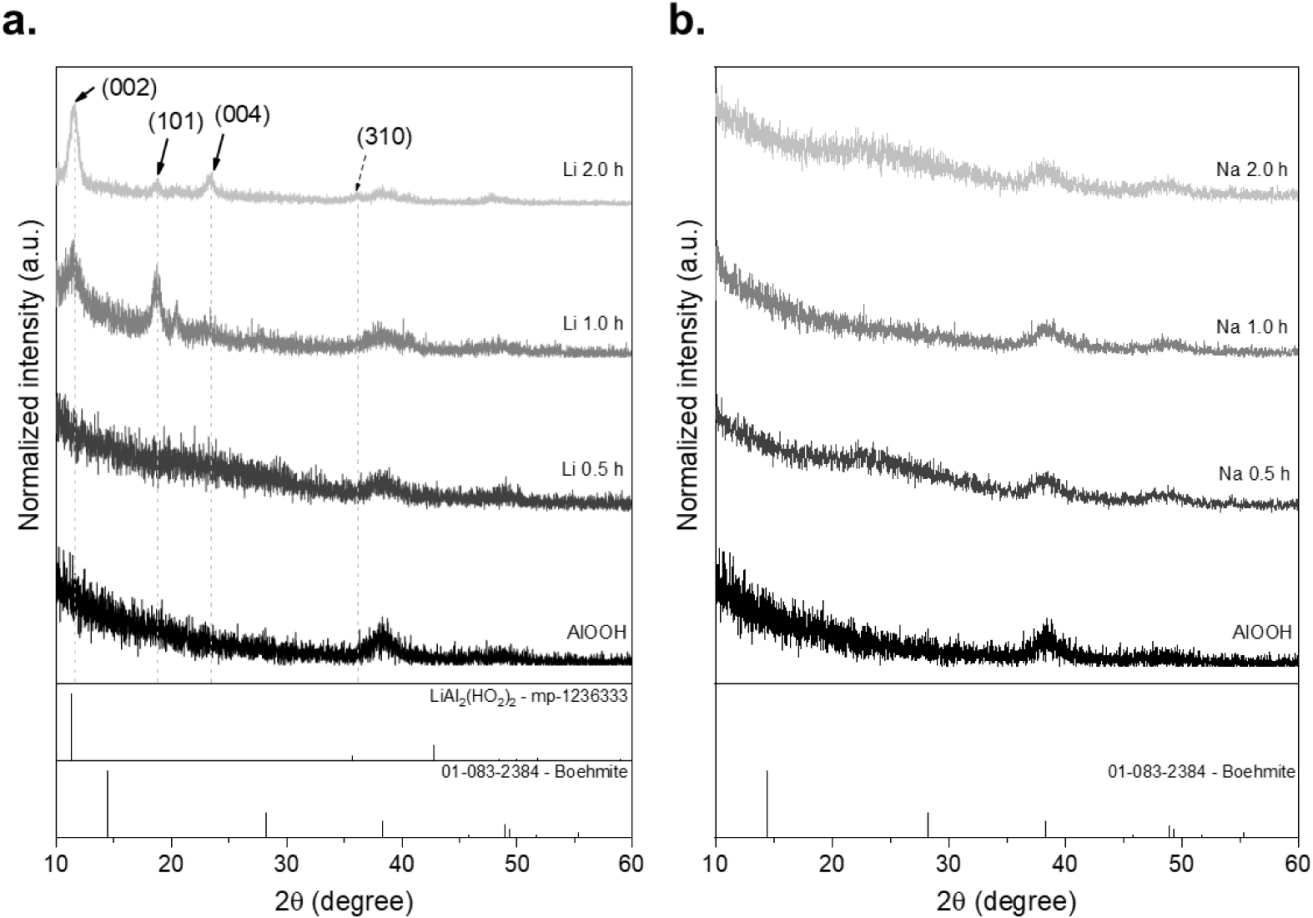
(a) XRD patterns of AlOOH films with Li^+^ and (b) XRD patterns of AlOOH films with Na^+^ at different immersion times.

In contrast, samples with the Na^+^ cation did not exhibit any prominent or characteristic peaks, as shown in Figure 2b, and what they yielded were the signals coming from AlOOH. However, it is not ruled out that a compound similar to that of the Li^+^ samples is occurring in these reactions; this may be attributed to variations in ionic radii and the interactions occurring between the lamellar planes of the AlOOH structure.

Although the synthesis was also performed by CBD methodology, no peaks related to any compound as a product were found, in addition to the boehmite phase not being found in the other diffractograms, as shown in Figure S1, because the film growth was accelerated, promoting the amorphousness of the material.

### 3.2 SEM Analysis

Analysis of the SEM micrographs reveals the effect that immersion caused on the surface of the AlOOH film presented above in Figure 1c at different immersion times. For the films with Li^+^, from Figure 3a–c, a growth is seen in the interconnected nanosheets. LiCl dissolution can significantly affect the growth and morphology of the AlOOH nanosheets. To understand these changes, it is necessary to analyze two fundamental aspects: the effect of the pH on boehmite formation and the impact of supersaturation. At high pH, nanoflake formation is favored due to the adsorption of OH^–^ groups.^32^ Upon immersion in LiCl solution, AlOOH films undergo a notable morphological and chemical transformation driven primarily by localized dissolution–reprecipitation (Ostwald ripening). This process promotes the dissolution of smaller crystallites and the redeposition of their material onto larger, preexisting nanoflakes, resulting in a transition from compact to more open and oriented lamellar structures. The partial solubility of AlOOH in the LiCl environment slightly reduces supersaturation, favoring this anisotropic growth rather than the formation of new nuclei.^33^ Concurrently, the intercalation of small Li^+^ ions facilitates the swelling and reorganization of the nanoflakes, enabling easier access of lithium and carbonate species into interlayer regions. This leads to the incorporation of secondary Li-aluminate or Li-carbonate phases within the boehmite matrix. The resulting expanded nanoflake architecture increases the effective surface area and reduces the ionic transport tortuosity, which correlates strongly with the observed enhancement in ionic conductivity. Such structural and chemical modifications are particularly advantageous for microbattery applications, where rapid ion transport and thin, efficient solid electrolytes are essential.

**Figure 3:**
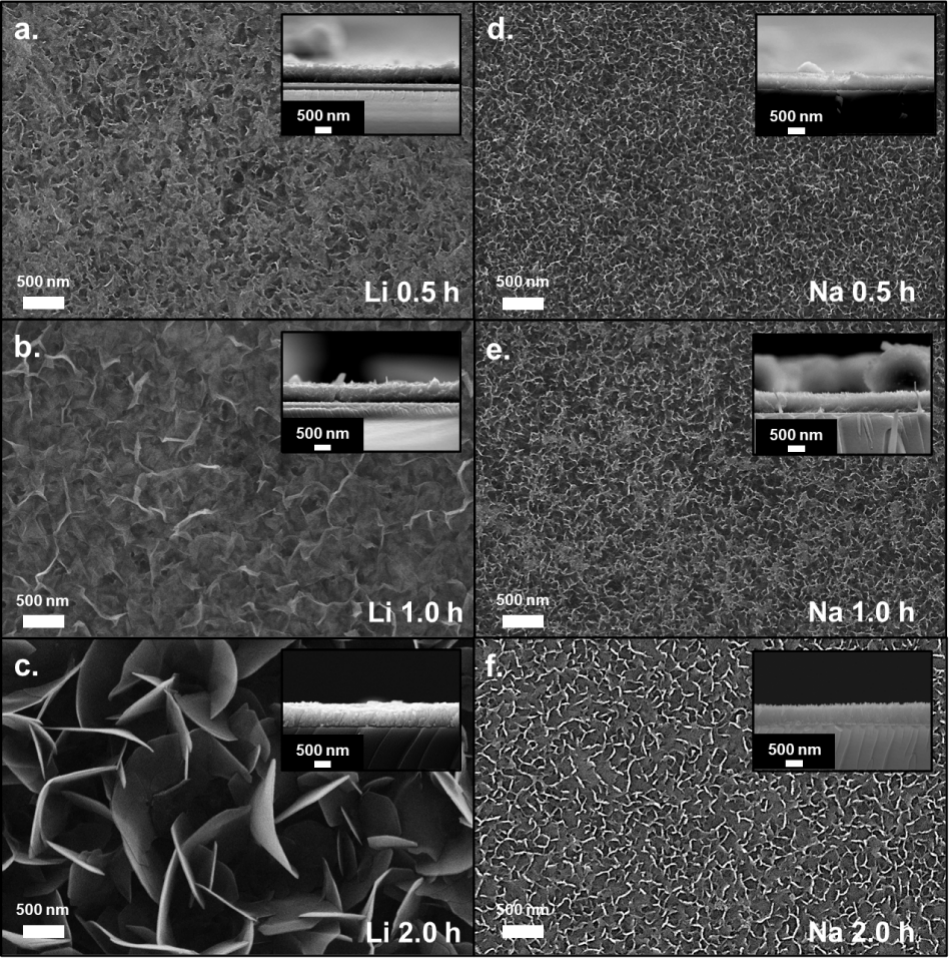
SEM images of (a–c) AlOOH films with Li^+^ and (d–f) films of AlOOH with Na^+^ with their respective cross-sectional images.

On the other hand, films with Na^+^ from Figure 3d–f are not as affected as those that went through an immersion process with LiCl. This could be because Li^+^ ions are smaller and have a charge density higher than that of Na^+^, which allows them to interact more strongly with AlOOH species in solution. Li^+^ ions have a greater tendency to adsorb on the AlOOH surface, modifying the surface charge.

In the case of determining the thickness of the films, SEM images in the cross-section were used, which yielded the following thicknesses: Li 0.5 h, 586 nm; Li 1.0 h, 602 nm; Li 2.0 h, 641 nm; Na 0.5 h, 581 nm; Na 1.0 h, 647 nm; and Na 2.0 h, 705 nm. The results indicated that although NaCl dissolution did not significantly modify the surface area of the films compared to LiCl, it did influence their thickness. This effect could be due to the adsorption of Na^+^ ions on the AlOOH surface, which partially neutralizes their charge and decreases the electrostatic repulsion between the nanosheets. Consequently, aggregation and compaction of the growing structures are facilitated, increasing the film thickness. Due to the lack of information on the growth of this material by chemical bath and its interaction with aqueous salt solutions, the available explanations are based solely on experimental observations.

### 3.3 XPS Analysis

In Figure 4a for the C 1s orbital, the presence of carbonates (CO_3_^2–^) is very evident when the immersion time is longer, while in Figure 4b, these CO_3_^2–^ are less present. The presence of surface carbon is apparent for both films in the first 0.5 h. However, an explanation for the films with longer immersion time could be because the higher carbonate accumulation in AlOOH films immersed in LiCl with respect to NaCl is due to the different interactions of Li^+^ and Na^+^ with the material surface, their influence on the local pH, and the differential solubility of AlOOH in both solutions. These combined factors make LiCl favor carbonate adsorption, whereas this process is less efficient in NaCl. The interaction of Li^+^ with hydroxyl groups (OH^–^) of AlOOH could generate surface sites with an affinity for CO_2_, favoring the formation of carbonates captured from the environment and generating carbonate species that can interact with the AlOOH film. These carbonates appear around 289.46 eV.^34–38^

**Figure 4:**
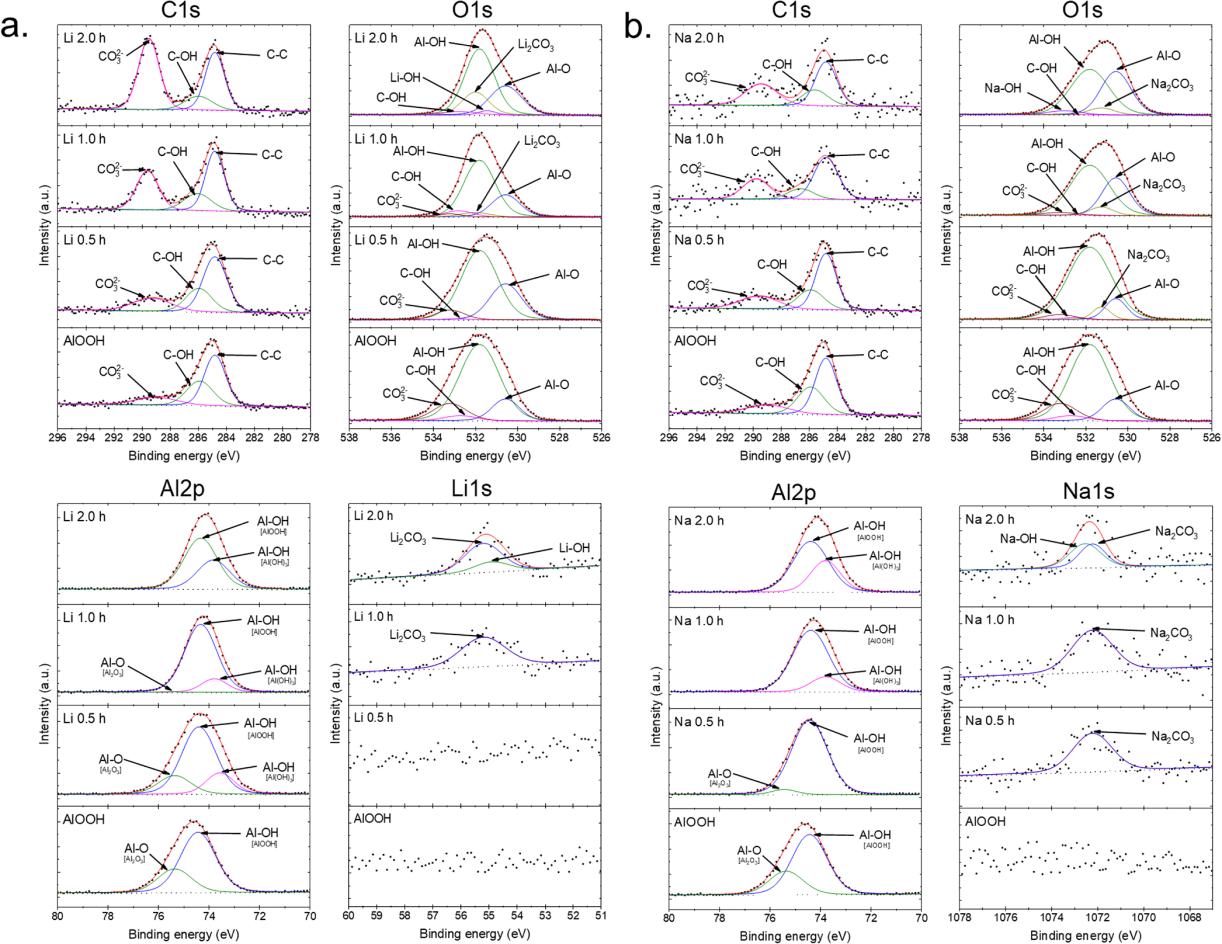
XPS spectra of (a) AlOOH films with Li^+^ ions and (b) AlOOH films with Na^+^ ions.

For the films with Li^+^, the O 1s orbital region in Figure 4a, a slant is observed. The longer the immersion time of the film, thanks to the contributions of Li–OH and Li_2_CO_3_ that are adhering to the AlOOH material, which appear around 531.43 and 532.00 eV, respectively,^34,39^ Li_2_CO_3_ being dominant due to ambient CO_3_^2–^. Likewise, an imbalance is observed in Al–OH [AlOOH] and Al–O [Al_2_O_3_] contributions with energies around 531.8 and 530.56 eV, respectively.^29–31,37,40,41^ Likewise, for the O 1s region of the films with Na^+^ in Figure 4b, however, in this case NaOH and Na_2_CO_3_ are present at around 533.00 and 531.24 eV, and its area under the curve increases as the immersion time increases.^37,42^

On the other hand, both films in the Al 2p orbital region, as shown in Figure 4a,b, share a decrease in the area of the Al–O contribution region and an appearance of Al–OH [Al­(OH)_3_] of a different chemical species, present around 73.78 eV, respectively,^29–31,37,40,41^ in addition to a shift toward low binding energies. This may indicate that the films in both salt solutions, when in direct contact with water for prolonged times, may rehydrate by further absorbing hydroxyls, causing the Al–O contribution from the oxide part of the AlOOH material to decrease and Al–OH with a different chemical environment to be added.

The different orbitals, such as Li 1s in Figure 4a and Na 1s in Figure 4b, present an intensity growth relationship; this is due to the immersion time of the films and the uptake work of the free Li^+^ and Na^+^ ions in the AlOOH films. For the Li 1s orbital, there are two contributions, those of Li–OH and Li_2_CO_3_ with positions around 54.88 and 55.18 eV, respectively.^34,39^ They are present in the O 1s orbital region. On the other hand, the same occurs in the Na 1s orbital region; in this case, Na–OH and Na_2_CO_3_ are present with positions around 1072.50 and 1072.19 eV, respectively.^37,43^

### 3.4 Electrical Analysis

The geometry used to measure these electrical and electrochemical properties was that presented in Figure 5a, in which square contacts were deposited using shadow masks on the analyzed films, with a contact spacing of 310 μm and a contact size of 240 μm. This architecture was used by the authors Hu et al.^14^ and Koh et al.^44^ The analysis of the data obtained by EIS was carried out using Gamry software through the construction of equivalent circuits that represent the phenomena occurring in the materials. The following elements were used to design these circuits: electrode resistance (R_s_), solid electrolyte interface resistance (R_SEI_), charge transfer resistance (R_ct_),^45^ constant phase element at the solid electrolyte interface (CPE_SEI_), constant phase element at the double layer (CPE_ct_),^46,47^ capacitance associated with charge transfer (C_ct_), intercalation capacitance (C_i_),^46^ and Warburg resistance (Z_W_). The combination of these components resulted in four different equivalent circuit configurations, shown in Figure 6a–d. Circuit I was used to fit the Nyquist curves of the Li^+^ films at temperatures from RT to 65 °C (Figure 6a,c); circuit II was used for the Nyquist curve of the Li^+^ film at 80 °C in Figure 6a; circuit III for the Nyquist curve of the Li^+^ film at 80 °C in Figure 6c; and circuit IV for all temperatures of the Na^+^ films depicted in Figure 6b,d.

**Figure 5:**
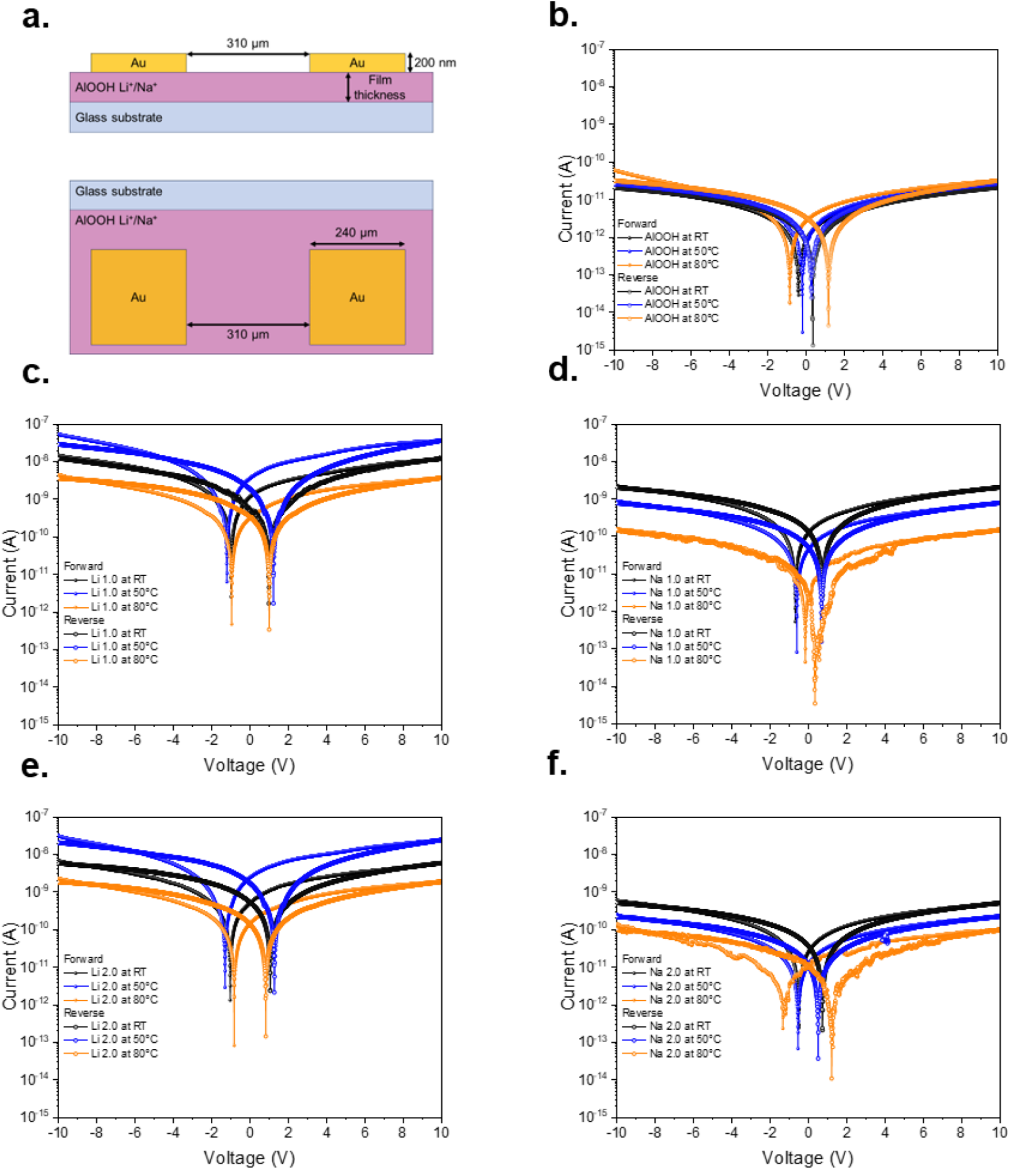
(a) Schematization of the geometry used for electrical and electrochemical measurements. Cross section (top) and top view (bottom). IV curves for (b) AlOOH film (as deposited), (c) Li 1.0 h film, (d) Na 1.0 h film, (e) Li 2.0 h film, and (f) Na 2.0 h film.

**Figure 6:**
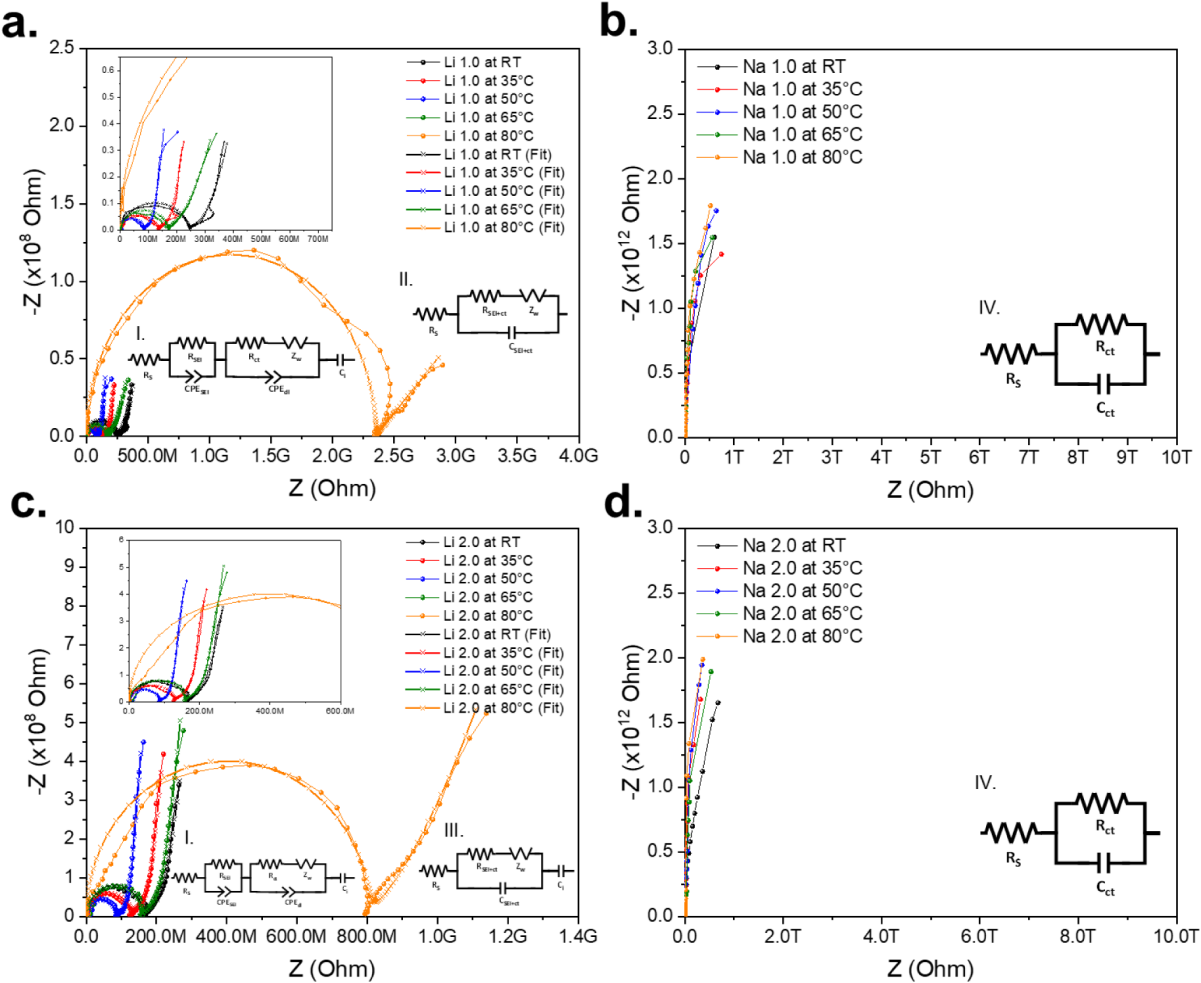
EIS Nyquist plot of (a) Li 1.0 h, (b) Na 1.0 h, (c) Li 2.0 h, and (d) Na 2.0 h films.

Figure 5b shows a typical IV curve of an AlOOH film measured from −10 to 10 V, where it can be noticed that the minimum value of the curve does not fall at zero voltage. This may be due to internal polarization caused by material defects in the bulk, such as hydroxyl (OH^–^) groups and oxygen vacancies.^29,48–51^ The shifts vary depending on the temperature at which they were measured, so other authors explain that there may be proton migration (H^+^) due to water adsorbed on the surface or due to the hydrated material itself.^52^ On the other hand, the current in negative and positive voltages was not affected by the temperature, remaining around 10^–10^–10^–11^ A.

From Figure 5c,e, it is observed that the AlOOH films with Li^+^ exhibit different behaviors compared to the AlOOH film used as a reference. At room temperature, the measured current is on the order of 10^–8^ A. However, by increasing the temperature to 50 °C, the current increases slightly for both samples. Interestingly, upon reaching 80 °C (Figure S2 shows the temperature sweep), a significant decrease in current, close to 10^–9^ A, is detected. This behavior could be attributed to ionic migration processes facilitated by the presence of Li^+^ and H^+^, whose mobility increases with temperature. This suggests that at higher temperatures, Li^+^ ions are more easily mobilized, increasing the ionic conductivity. At this temperature, there may be a more effective interaction between Li^+^ and the surface hydroxyl groups. This could enhance the transport of ionic charges. However, at 80 °C, the current decreases again, indicating that the material becomes more resistive at higher temperatures. This could be caused by the formation of a carbonate layer: if the film is interacting with CO_2_ from the air, lithium carbonates could form at high temperatures, and this would reduce the ionic conductivity.^53^ It becomes evident and reflects the same behavior by the Nyquist EIS technique in Figure 6a,c. Warburg resistance is associated with ionic diffusion processes and manifests itself before the linear section observed at low frequencies. In Figure 6a,c, this phenomenon is evident: after the end of the characteristic semicircle, a slope close to 45° is present, corresponding to Li^+^ ion diffusion. At even lower frequencies, the behavior becomes predominantly capacitive, as evidenced by an almost vertical line. According to Teo et al.,^46^ this capacitive regime may be due to the accumulation of Li^+^ ions at the interface, which hinders their mobility. However, it is also possible that it is related to the formation of lithium carbonate,^54^ which would limit ion migration.

The ionic conductivity values reported in Table 1 exhibit an increasing trend with increasing temperature, reaching a maximum at around 50 °C, followed by a decrease at higher temperatures, suggesting that ion mobility is restricted within a specific thermal range. At room temperature, the material exhibits an ionic conductivity of 0.77 × 10^–4^ S cm^–1^, which falls within the typical range reported for similar materials. For instance, Yi et al. reported comparable conductivities in the order of 10^–5^ to 10^–4^ S cm^–1^ at room temperature, although their samples were prepared using a sintering method.^55–57^ In the Nyquist curves between room temperature and 65 °C (Figure 6a,c), the semicircle is attributed to the formation of an interface between the electrode and the film.^47,54^ As the temperature increases, the resistance associated with this interface decreases until it reaches an inflection point, where it increases again. In particular, for films treated with Li^+^ at 80 °C, a noticeable increase in interfacial resistance is observed, possibly due to the accumulation of lithium carbonate.

**Table 1:** EIS Parameters of the Data Fitted to the Equivalent Electrical Circuit for Li^+^ Films^a^

Li 1.0 h							Li 2.0 h				
		RT	35 °C	50 °C	65 °C	80 °C	RT	35 °C	50 °C	65 °C	**80 °C**
R_SEI_	×10^8^ Ω	2.46	1.38	0.85	1.72		1.66	1.30	0.87	1.60	
CPE_SEI_	×10^–12^ Ω^–1^s^a^	1.75	2.45	10.59	2.07		1.93	14.94	5.21	2.55	
a_SEI_		**0.88**	**0.84**	**1.00**	**0.90**		**0.97**	**0.96**	**1.00**	**1.00**	
R_ct_	×10^8^ Ω	0.28	0.23	0.16	0.23	23.50	0.30	0.22	0.15	0.26	8.01
CPE_dl_	×10^–9^ Ω^–1^s^a^	3.68	6.50	7.01	5.00		7.44	8.32	3.21	3.85	
a_dl_		**0.87**	**1.00**	**1.00**	**1.00**		**0.90**	**0.90**	**1.00**	**1.00**	
C_ct_	×10^–12^ F					1.00					1.82
Z_w_	×10^–9^ Ω·s^1/2^	24.91	45.29	49.76	20.75	5.55	34.40	49.23	52.00	32.75	9.16
C_i_	×10^–8^ F	7.54	0.65	4.98	7.92		5.95	5.09	4.35	3.81	7.05
σ_in plane_	×10^–4^ S cm^–1^	0.77	0.93	1.34	0.93	0.01	0.67	0.92	1.34	0.78	0.03

a Note: In the case of the system resistance (Rs), a value close to 0 Ω was obtained. This result can be attributed to the in-plane configuration used in the measurements, where the resistance of the glass substrate and metal electrodes is negligible.

In contrast to what was observed for the Li^+^ samples and the AlOOH reference, Figure 5d,f reveals that the AlOOH films with Na^+^ films exhibit a different behavior: their electrical resistance increases with temperature. Although they do not reach the resistivity of the pure AlOOH film, the current decreases from about 1 × 10^–8^ A at 25 °C to about 1 × 10^–10^ A at 80 °C. This effect could be due to the lower ability of Na^+^ to intercalate into the AlOOH structure, attributed to its larger ionic radius compared to Li^+^, which would limit its mobility and favor the formation of regions where Na^+^ ions are trapped. The observed Nyquist plots in Figure 6b,d reflect the result of the IV curves, which is due to a highly capacitive behavior without effective charge transport.^58,59^ This behavior can be attributed to the immobilization of Na^+^ ions in the material structure or to the obstruction of the ionic conduction pathways, causing the system to function as a dielectric with charge accumulation at the interface. The parameters obtained from the fit with the equivalent circuit, presented in Table 2, do not show significant variations or a clear trend, indicating low ionic mobility under these conditions.

**Table 2:** EIS Parameters of the Data Fitted to the Equivalent Electrical Circuit for Na^+^ Films^a^

Na 1.0 h							Na 2.0 h				
		RT	35 °C	50 °C	65 °C	80 °C	RT	35 °C	50 °C	65 °C	80 °C
R_ct_	×10^12^ Ω	20.19	5.78	5.46	8.49	7.84	18.28	3.28	4.36	7.31	6.54
C_ct_	×10^–12^ F	11.14	9.13	7.57	9.36	8.33	22.13	8.23	6.67	9.01	9.33
σ_in plane_	×10^–9^ S cm^–1^	0.10	0.35	0.37	0.24	0.25	0.10	0.56	0.42	0.25	0.28

a Note: In the case of the system resistance (Rs), a value close to 0 Ω was obtained. This result can be attributed to the in-plane configuration used in the measurements, where the resistance of the glass substrate and metal electrodes is negligible.

## 4 Conclusions

The deposited AlOOH film presented the boehmite phase, confirmed by XRD and FTIR analysis, and showed good thermal stability with constant currents in IV scans of up to ± 10 V over the entire temperature range evaluated. Treatment with LiCl solution generated morphological changes that could be observed by SEM and allowed the incorporation of lithium, mostly in the form of carbonate, as evidenced by XRD and XPS. IV curves and EIS spectra evidenced a marked sensitivity to Li^+^ ion movement, as well as a strong dependence of ionic transport on temperature. At room temperature, the ionic conductivity was 0.77 × 10^–4^ S cm^–1^, increasing notably to 1.34 × 10^–4^ S cm^–1^ at 50 °C. In contrast, the NaCl-treated films retained their structure and morphology without significant changes and exhibited an increase in resistivity with increasing temperature, indicating a limited capacity for ionic conduction, remaining around ×10^–9^ S cm^–1^. Taken together, these findings indicate that lithium incorporation significantly improves ionic transport, making these films promising candidates for energy storage applications.

## Supplementary Material



## Notes

X-ray diffraction of AlOOH films incorporated with Li^+^ and Na^+^ ions obtained by the chemical bath deposition method; measurements of current–voltage (IV) curves performed on the reference (AlOOH) and modified with Li^+^ and Na^+^ films, evaluated at different temperatures: ambient, 35, 50, 65, and 80 °C (PDF)
