# Supplementary material for: Intercalation of Li + vs Na + Ions in AlOOH Thin Films: Toward Low-Cost Solid-State Electrolytes
Source: ACS Omega. 2025 Jul 21;10(30):33767–76. doi: 10.1021/acsomega.5c04820 (PMC12332659; doi:10.1021/acsomega.5c04820)
Supplement: Supplementary file 1 [file ao5c04820_si_001.pdf]

Intercalation of Li^+ vs Na^+ ions in AlOOH thin films: Toward low-cost solid-state electrolytes

Martin A. Ruiz-Molina [a,b], Leunam Fernandez-Izquierdo [b], Ruben O. Grijalva-Saavedra[a], Manuel Quevedo-Lopez[b], Merida Sotelo-Lerma [a,b,*]

[a] Department of Research in Polymers and Materials, Universidad de Sonora, Blvd. Luis Encinas y Rosales S/N, Hermosillo 83000, Sonora, Mexico

[b] Materials Science and Engineering Department, University of Texas at Dallas, 2601 North Floyd Road, RL 10, Richardson, TX, 75080, USA.

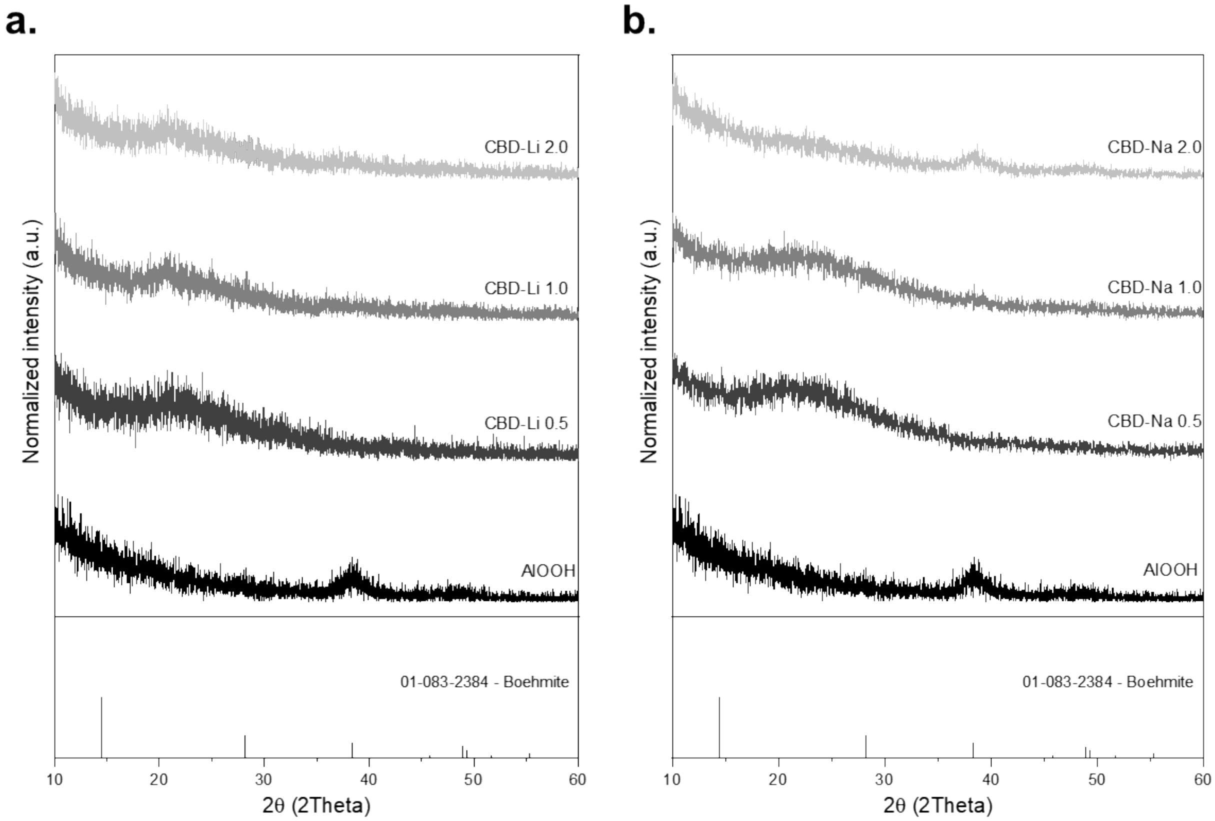

Figure S1 a. XRD patterns of AlOOH films with Li^{+} and **b.** XRD patterns of AlOOH films with Na^{+} by chemical bath deposition at different deposition times.

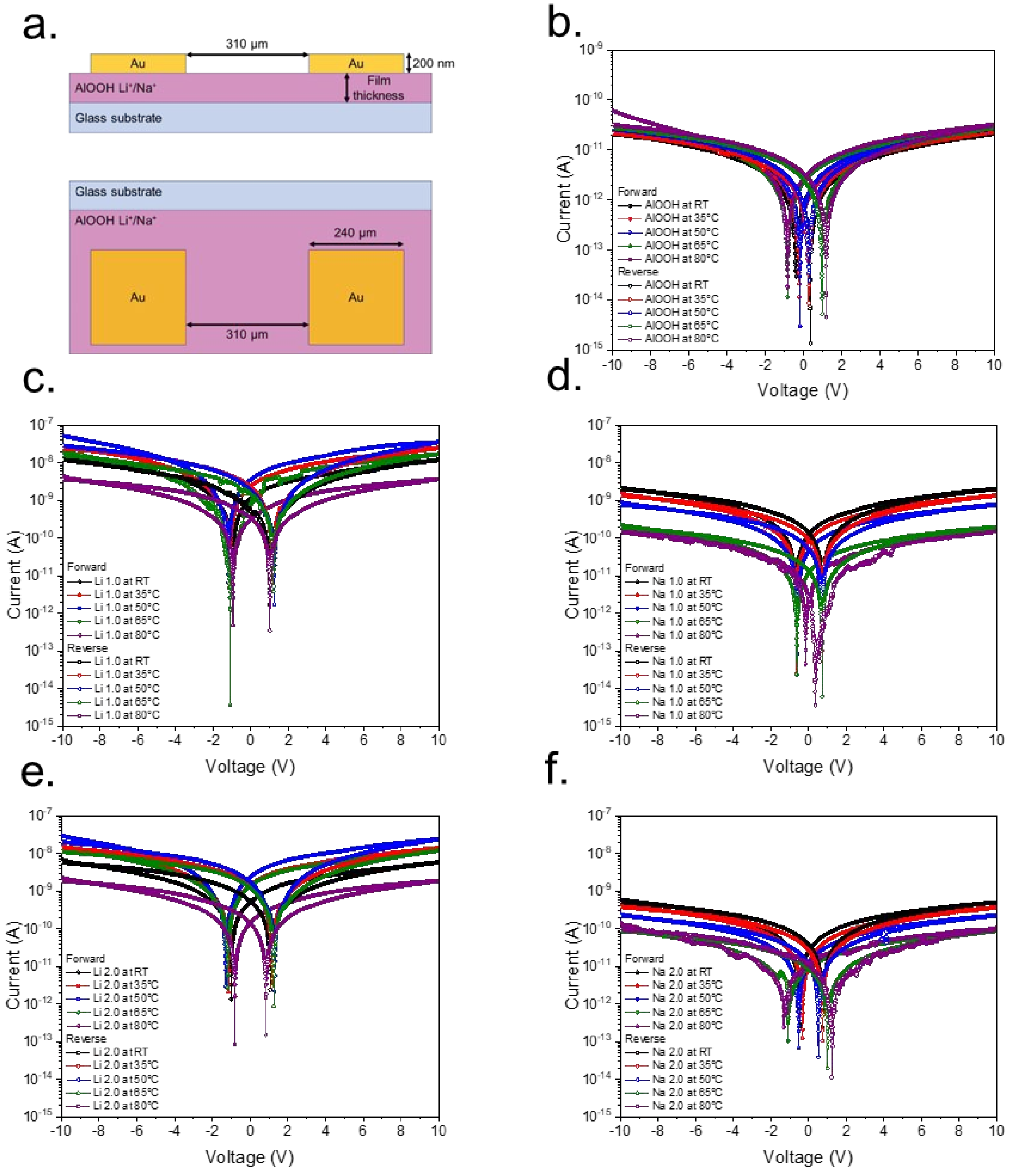

Figure S2 a. Schematization of the geometry used for electrical and electrochemical measurements. Cross section (top) and top view (bottom). IV curves for; **b.** AlOOH film (as deposited), **c.** Li 1.0 h film, **d.** Na 1.0 h film, **e.** Li 2.0 h and **f.** Na 2.0 h.
